# Outcome of a research ethics training workshop among clinicians and scientists in a Nigerian university

**DOI:** 10.1186/1472-6939-9-1

**Published:** 2008-01-24

**Authors:** Ademola J Ajuwon, Nancy Kass

**Affiliations:** 1 African Regional Health Education Center, Department of Health Promotion and Education, College of Medicine, University of Ibadan, Nigeria; 2 Department of Health Policy and Management, Bloomberg School of Public Health, Berman Bioethics Institute, Johns Hopkins University, Baltimore, Maryland, USA

## Abstract

**Background:**

In Nigeria, as in other developing countries, access to training in research ethics is limited, due to weak social, economic, and health infrastructure. The project described in this article was designed to develop the capacity of academic staff of the College of Medicine, University of Ibadan, Nigeria to conduct ethically acceptable research involving human participants.

**Methods:**

Three in-depth interviews and one focus group discussion were conducted to assess the training needs of participants. A research ethics training workshop was then conducted with College of Medicine faculty. A 23-item questionnaire that assessed knowledge of research ethics, application of principles of ethics, operations of the Institutional Review Board (IRB) and ethics reasoning was developed to be a pre-post test evaluation of the training workshop. Ninety-seven workshop participants completed the questionnaire before and after the workshop; 59 of them completed a second post-test questionnaire one month after the workshop.

**Results:**

The trainees came from a multi-disciplinary background including medicine, nursing, pharmacy, social science and laboratory science. The mean scores for knowledge of the principles of research ethics rose from 0.67 out of 3 points at pre-test to 2.25 at post-test (p < 0.05). Also, 42% correctly mentioned one international guideline or regulation at pretest, with most of those knowing of the Declaration of Helsinki. Trainees' knowledge of the operations of an IRB increased from 6.05 at pre-test to 6.29 at post test out of 7 points. Overall, participants retained much of the knowledge acquired from the workshop one month after its completion.

**Conclusion:**

The training improved participants' knowledge of principles of research ethics, international guidelines and regulations and operations of IRBs. It thus provided an opportunity for research ethics capacity development among academic staff in a developing country institution.

## Background

Research ethics in its broadest definition encompasses the principles, standards, norms and guidelines that regulate scientific inquiry [1]. The primary role of ethics in health research is to protect the rights, integrity, and safety of research participants. Public awareness of abuses to human research participants, including the horrific human experiments of the Second World War in Germany and the Tuskegee syphilis study in America, led to the formulation of several ethical guidelines including the Nuremberg Code, the Declaration of Helsinki, and the Council for International Organizations for Medical Sciences (CIOMS) guidelines.

Despite the availability of these guidelines and regulations, violations of the rights of research participants continue to occur in both higher and lower income countries [1-5]. It has been suggested also that African study participants are more susceptible than their counterparts in developed countries to exploitation because of high levels of poverty, low literacy rates [6], severely limited access to basic health care [7], and inadequate local regulation of biomedical research [8]. Recent research ethics controversies in Africa include the Pfizer drug trials of *Trovan *in Nigeria and *Tenofovir *in Cameroon that have again highlighted the need for African professionals to have sophistication in research ethics in order to be able to participate in the debates locally [8].

One of the strategies for addressing this situation is initial and continuing education in the ethics and science of biomedical and behavioral research for investigators, members of the Institutional Review Boards (IRBs), and sponsors of research [9,10]. Training has three roles to play in ensuring the protection, safety, and integrity of study participants. First, formal and educational updates in research ethics can help increase professionals' knowledge and sensitivity to new and emerging ethical concerns in the conduct of research. For example, training and continuing education sensitize scientists to the ethical issues arising from rapid advances in medicine and biotechnology such as research into genome, stem cells, multi-country field trials and experimentations involving human vulnerable populations [11]. Second, training has been shown in some settings to be effective in providing scientists with skills for dealing with the ethical dilemmas they encounter in their own research. In a training program for American surgical residents, Pollock and colleagues found that trainees in research ethics were better able than non-trainees to deal with problems relating to how to proceed if they lacked a sufficient quantity of a reagent critical for experimental data replication, and if they had problems with discordant or outlier experimental data. Trainees were prepared to seek third party input for resolving a dilemma involving their own work [12]. A study by Brown and Kalichman [13] among graduate students in experimental sciences also showed that training resulted in improved reports of knowing what to do if faced with an ethical dilemma. Finally, training in research ethics affords scientists, especially those from developing countries, the opportunity to contribute to ever increasing international debates on ethical issues [6], many of which are likely to take place in developing countries.

In Nigeria, as in other developing countries, the limited access to training in research ethics is due to weak social, economic, and health infrastructures. Although two national research ethics training programs have been conducted in Nigeria during the last five years, these remain inadequate to meet the ever growing needs of scientists [14]. In addition, none of the previous programs have included a systematic evaluation of their outcomes. The project described in this article was designed to address this gap. This paper describes the processes and outcomes of a research ethics training workshop and evaluation conducted for academic staff of the College of Medicine, University of Ibadan, Nigeria. Funding was obtained from the Fogarty International Center, National Institutes of Health, USA and the Wellcome Trust, UK to support the workshop and evaluation reported here.

## Methods

This project was approved by the IRBs of the University of Ibadan/University College Hospital Ibadan and the Bloomberg School of Public Health, Johns Hopkins University, Baltimore, Maryland, USA.

### The setting

The University of Ibadan (UI), Nigeria was established in 1948 and is the oldest institution for tertiary education in the country. The College of Medicine (CM) is a semi-autonomous branch of UI affiliated with the University College Hospital (UCH), a teaching hospital that provides tertiary care and trains different categories of health workers, including physicians, dentists, nurses, and laboratory scientists. The CM has 32 departments/units spread into four Faculties: Basic Medical Sciences, Clinical Sciences, Dentistry, and Public Health. As of September 2003, when the study began, the CM had approximately 250 academic staff including physicians, dentists, nurses, laboratory scientists, and public health professionals.

#### Needs assessment

The first step in planning this study was to conduct needs assessment to identify appropriate content and structure of the training. To this end, in-depth interviews were conducted with the Chair and Co-Chair of the IRB, and the administrator of the institution. An in-depth interview guide was developed and used for data collection. In addition, one focus group discussion (FGD) session, which lasted approximately one hour thirty minutes, was conducted with eight of the 15 members of the College's IRB. The first author moderated the focus group discussion with secretarial assistance provided by a trained project officer. The interview and discussion guides explored three themes: challenges to quality ethics review, good ethical consideration, identification of content for the proposed ethics training, and suggestions for its successful implementation. After a discussion of problems relating to good ethical consideration, each FGD participant was given a list containing nine topics covering a broad spectrum of issues. These topics were derived from the research ethics training curriculum developed by Family Health International (FHI) [15]. Each FGD participant was requested to rank three topics in order of their perceived priority. The list included principles of research ethics, international regulations and guidelines, informed consent, IRBs, ethics and research designs, standard of care, justice/obligations of researchers to research participants, conflict of interest and scientific misconduct. Both the interviews and discussions were recorded on audio-tapes.

Participants identified five problems hindering quality ethics review: delay in review of proposals by the local IRB, weak monitoring system of approved proposals, lack of training of some members of the IRB, a misunderstanding of the role of an IRB, and lack of understanding by many scientists of informed consent procedures. One interviewee attributed the inadequate understanding of informed consent process to "*the master-servant relationship between physicians and patients in this environment*" and the fact that research ethics is not included in the curriculum for medical education. Regarding scientists' lack of understanding of the role of an IRB, one respondent said that "*Many scientists still do not fully understand the role an ethics committee ought to play in a research project. Some believe that members of the committee will plagiarize their ideas during the review process" *and are therefore reluctant to submit proposals for review. FGD participants perceived that the delay in the proposal review process was the most important problem affecting both the IRB and investigators.

There was consensus of opinion among interviewees and FGD participants that the proposed training on research ethics was both timely and appropriate. As one interviewee pointed out, "*The workshop (training) would create a lot of awareness and this awareness would have a ripple effect and ultimately improve the quality of protocols submitted to the IRB for review." *One of the interviewees also stated that, "t*he workshop would serve as a stimulant. It would also create awareness. It would stimulate people on why research ethics is important. It would also open their [investigators] eyes to the role of the IRB."*

FGD participants ranked principles of research ethics as the most appropriate content, followed by international guidelines, informed consent, ethics of research design, justice and obligation, scientific misconduct, and IRBs, in that order. None of the participants considered standard of care, obligations of researchers to study participants and conflict of interest to be relevant content for training.

To ensure the success of the training, it was suggested that there was a need to invite those who have genuine interest in learning about research ethics, that adequate publicity should be provided for the training, and that resident doctors and other post-graduate students should be invited to participate. There was also a suggestion that case studies and proposal writing should be included in the workshop.

These findings were considered in the planning and implementing of the training program.

### Recruitment of trainees

In order to ensure wide participation of staff in the training, a letter was written to the Heads of all the 32 departments/units in the institution to solicit nominations from each. The number of staff was limited to four per department or unit due to funding constraints. The letter of invitation stressed the fact that participation in the program was voluntary. Of the 32 departments/units invited to nominate staff, 29 (91%) responded and 3 (9%) did not. In addition, two Non-Governmental Organizations that routinely conduct behavioral and biomedical researchers in Ibadan were invited to nominate staff to participate in the training. Upon receiving names of nominees, a formal letter of invitation was sent to each person providing details about the logistic arrangements for the training.

### Implementation of the workshops

Three rounds of training workshops were conducted between December 2003 and February 2004. Each round, which lasted 21 hours spread over three days, was organized in ways that ensured that the composition of the trainees was multi-disciplinary. The objectives of the workshop were to expose participants to existing international guidelines and regulations on research involving human participants, increase knowledge and application of the principles of research ethics, deepen understanding of the role of investigators and IRBs in ensuring the protection of study participants, and equip participants with knowledge and skills to deal with the ethical challenges they may face in their future research activities. The workshops were facilitated by seven resource persons who had previous training and experience in research ethics. The resource persons used multiple training techniques including lecture, question and answer sessions, and group discussion of case studies. The training had both plenary and group sessions. Participants were divided into small groups to discuss the case studies and feedbacks were provided during plenary sessions thus ensuring in-depth discussions of the issues. The workshops were conducted in English, the official language in Nigeria. The training emphasized the importance and universality of the principles of research ethics as described in the Belmont Report; however, the resource persons provided several examples of the local application of these principles. The contents covered during the workshop were history of research ethics, principles of research ethics, ethics in research design, ethical issues in drug/vaccine trials, confidentiality\obligation, informed consent, conflicts of interest and scientific misconduct. Twenty reference materials were distributed and certificates of attendance were presented.

### Measures

Knowledge measures were administered pre-test, on the morning of the first workshop date, post-test at the end of the last workshop afternoon, and then also one month after the workshop was completed. The questionnaire consisted of 23 open and closed-ended items covering demographic characteristics, knowledge of international guidelines, the principles of research ethics and their application, and operations of IRBs. The questionnaire was an adaptation of the instrument developed by the FHI for its internet-based research ethics training program [15]. Knowledge was assessed by requesting trainees to list three international guidelines and regulations that they knew, and identify three issues that they perceived were important for the conduct of such research. In addition, six true/false statements were formulated for trainee response. Examples of the statements read, "*Only research funded by an external agency should be submitted to the Ethical Review Committee for consideration*," and, *"approvals from an Ethical Review Committee are for the duration of the research*." Trainees were also requested to identify the principle of research ethics that matched each of a different set of seven statements. A sample statement read, "*The special needs of vulnerable populations such as prisoners and children must be protected at all times" *(see Additional File 1 for details).

To assess participants' level of ethics reasoning, two case studies developed by the investigators were included in the questionnaire. The case studies focused on conflicts of interest and obligations of investigators to research participants (see Additional File 1). After reading the case studies, trainees were requested to respond to four statements with "agree" or "disagree" options. The questionnaire was pre-tested for comprehension and clarity among five academic staff of the Faculty of Social Sciences of the same university. It was assumed that voluntary return of the completed questionnaire indicated informed consent.

### Data analysis

The audio-tapes of recorded interviews and discussions for the needs assessment were played, transcribed and themes were developed. For the pre-test and post-tests, questionnaires were collated, open-ended questions on the questionnaire were coded, and data were entered into a computer. Analysis was performed using the EPI-Info 6.04 software package developed by the United States Centers for Disease Control and Prevention Atlanta, Georgia, USA. The data were analyzed and presented to reflect three points in time: before, immediately after, and one-month following the workshop. The responses concerning knowledge about principles of research ethics (question 5) are presented both in percentages and mean scores. The first process in analyzing the answers to this question was to enumerate all the responses mentioned by trainees. Codes were then assigned to each answer indicating whether the response corresponded with any of the three principles. In computing the mean score, one point was assigned for each correct answer such that three possible points could be achieved for this knowledge score.

We also developed a seven-point knowledge score in assessing trainees' knowledge of the application of principles of research ethics (questions 8–14). Similar procedures were adopted for knowledge about operations of an IRB (question 7). Using a chi-square test, we compared mean values of the trainees on each component of knowledge variable at three points in time, before, after and follow-up.

## Results

### Response Rate

Of the 133 persons who attended the workshops, 97 (73%) completed the pre-test and post-test questionnaire. Of the 97 trainees contacted at one-month follow-up, only 59 (61%) returned their questionnaire. Thirty-six trainees did not complete the pre-test because they either arrived late (i.e. after the pre-test had been conducted) or did not provide informed consent. There is no data on the demographic characteristics of persons who did not complete the pre-test. Those who did not complete the pre-test were told they were not eligible for the post-test or follow-up survey.

### Profile of trainees

The 97 trainees came from multi-disciplinary backgrounds including medicine, nursing, pharmacy, social science and laboratory science (Table 1). Their ages ranged from 28 years to 68 years with a mean of 41.7 years. There were slightly more males (51%) than females (49%). Ten trainees (10.3%) were members of the local IRB; 34% of all participants reported that they had had a previous training on research ethics, and 64% had none.

**Table 1:** Demographic Profile of trainees on research ethics (N = 97)

**Affiliation of participants**	**N**	**(%)**
Clinical Science	37	38.1
Basic Medical Science	18	18.6
Public Health	17	17.5
Dentistry	9	9.3
Non-Governmental Organization	5	5.2
Education	3	3.1
Social Science	2	2.0
Pharmacy	1	1.0
Law	1	1.0
NA	3	3.1
**Years of research experience**		
1–5	27	29.3
6–10	27	29.3
11–15	17	18.6
16–20	12	13.0
21–25	5	5.4
26–31	4	4.3
**Qualifications**		
MBBS/BDS	8	8.2
M.SC/MPH	35	36.1
PhD/MD	15	15.5
FNCP, FWCP, FNCPH	37	38.1
Diploma in Community Development	2	2.1

### Knowledge of principles of research ethics and guidelines

Trainees were requested to list the three most important considerations that should guide the conduct of research involving human participants. At baseline (pre-test), 35.1% of trainees provided answers relating to beneficence, 22.7% mentioned justice, but no one provided answers relating to respect for persons. Examples of answers that were not directly related to the three principles of ethics include, "conduct ethically sound research," "non-personalizing issues" "non-exploitation," and "notification of side effects." By post-test, 97% provided answers relating to beneficence, 68% mentioned justice, and 68% invoked the concept of respect for persons. The mean scores for knowledge of the principles of research ethics rose from 0.67 out of 3 points to 2.25 at post-test; this dropped to 2.19 at follow-up (p < 0.05).

On the topic of awareness of international guidelines or regulations, 42% correctly mentioned at least one known guideline at pre-test; 58% could not list any. Of those who knew of a guideline, 71% listed the Declaration of Helsinki, 20% mentioned the Nuremberg Code and 9% identified the Belmont Report. None of the participants mentioned CIOMS at pre-test. The proportion that mentioned these guidelines at post-test were 67.0%, 42.3%, 33.0%, 37.1% respectively.

### Application of the principles of research ethics

The proportion of trainees who provided correct answers before, after, and at 1-month follow-up to the seven statements that measured application of research ethics is shown in Table 2. Overall, trainees' knowledge improved at post-test on five of the seven statements. At pre-test, 65% knew that the principle of respect for persons was applicable to the statement, "*the capacity and rights of all potential Nigerian research participants to make their own decision must be respected by all investigators*." This figure rose to 87% at post-test, but dropped slightly to 81% at follow-up. Similarly, at pre-test, 43% of trainees knew that the principle of justice was applicable to the statement, "*the use of poor Nigerians for the exclusive benefit of more privileged Nigerians should be discouraged*." This figure rose to 60% at post-test, and was 59% at follow-up. When the scores for the seven items were summarized, the mean rose from 2.64 at pre-test to 3.05 and 3.24 at post-test and follow-up respectively (p < 0.05).

**Table 2:** Percentage of trainees providing correct answers to questions on the applications of the principles research ethics

Statements	Pre-test (N = 97)	Post test (N = 97)	Follow-Up (N = 59)
1. The capacity and rights of all potential Nigerian research participants to make their own decision must be respected by all investigators (respect for persons)	65	87	81.4
2. The special needs of vulnerable populations such as prisoners and children must be protected at all times (beneficence)	30	35	35.6
3. The protection of the Nigerian research participants is more important than the pursuit of new Knowledge by scientists (respect for persons).	31	16	23.7
4. It is the responsibility of researchers to maximize benefit and minimize risk of all persons who take part in a research (beneficence)	53	68	69.5
5. All segments of the Nigerian population must be fairly selected as participants in any Research project (Justice)	11	22	23.7
6. The use of poor Nigerian research participants for the exclusive benefit of more privileged Nigerians should be discouraged (Justice).	43	60	59.3
7. Nigerian investigators have the responsibility of ensuring the physical, mental and social well being of all Nigerians who volunteer to take part in any research project (beneficence)	32	19	30.5

### Knowledge about operations of an IRB

Table 3 shows trainees' knowledge of the operations of an IRB at three points in time. Generally, trainees had high levels of knowledge on all the relevant items at the three points in time. For example, virtually all the trainees – (99%) pre-test, (100%) post-test and follow-up – knew that this statement was false: "Only research funded by external agency should be submitted to the ERC for consideration." Similar findings were observed for the statement, "only members of an institution can be appointed to serve in ERC" (92% correct at pre-test, 100% correct for both post-test and follow-up). However, one exception to this trend was found in responses to statement number 4 which says that, *"IRB approvals of a research project are for the duration of the project." *Only 27% of trainees answered correctly that this is not true, indicating that they appeared to recognize the concept of "expiration" and/or the process of "renewal." This proportion rose to 57% at post-test, but dipped to 40.7% at follow-up. Additional details are provided in Table 3. The score on this variable was summarized and it was found that at pre-test, the mean score of this variable was 6.05 out of 7 points; this number rose to 6.29 at post-test but reduced to 6.27 at follow-up (p < 0.05).

**Table 3:** Percentage of trainees providing correct answers to questions on the operations of IRB

**Statements.**	**Pre-test** **(N = 97)**	**Post test** **(N = 97)**	**Follow-up** **(N = 59)**
1. Only research funded by an external agency should be submitted to the Ethical Review Committee for consideration (False).	99	100	100
2. Once they are enrolled, participants in a research cannot withdraw from the study without prior approval or agreement from the principal investigator (False).	92	94	100
3. The information in a consent form must be presented in a manner that is comprehensible to the research participants (True).	99	93	98.3
4. Approvals from an Ethical Review Committee are for the duration of the research (False).	27	57	40.7
5. It is the responsibility of the researcher to develop a scientifically sound research protocol (True).	99	97	98.3
6. The main function of an Ethical Review Committee is to ensure the protection of participants who volunteer to take part in a research (True)	90	84	89.8
7. Only members of an institution can be appointed into Ethical Review Committee (False)	92	100	100

### Ethics reasoning in case studies

Tables 4 and 5 show participants' responses to the statements listed in the case study on conflict of interest. At pre-test, 76% correctly "agreed" that Madral had a conflict of interest; 95% of post-test respondents recognized Madral's conflict, and 88% did so one month later. Those who "disagreed" with the statement that "patients recruited into the study will definitely benefit from the new drug" rose from 74% at pre-test to 86% at post test and dropped to 81% at follow-up.

**Table 4:** Trainee's attitude to ethical issues in case study on conflict of interest

Statements	Pre Test (%) (N = 97)		Post Test (%) (N = 97)		Follow-up (%) (N = 59)	
	
	Agreed	Disagreed	Agreed	Disagree	Agreed	Disagree
Madral has a conflict of interest	76	24	95	5	88	12
The study was not reviewed by an ethics review committee	83	17	86	14	78	22
The amount paid to patients is acceptable or adequate	25	75	44	56	44	56
Madral may experience some tension between his interests as a scientists and his interest as a share holder in the company that sponsored the research	90	9	98	2	98	2

**Table 5:** Trainees' attitude to statements in case study on conflict of interest

Statements	Pre Test (%) (N = 97)		Post Test (%) (N = 97)		Follow-up (%) (N = 59)	
	
	Agreed	Disagreed	Agreed	Disagree	Agreed	Disagree
Patients recruited into this study will definitely benefit from the new drug.	20	74	13	86	19	81
Patients who participated in this study will definitely have access to the drug if proven to be effective	28	70	16	84	15	85
The amount being paid to participants as compensation is acceptable or adequate	24	71	42	57	36	64
Madral may experience some tension between his interests as a scientists and his interest as a share holder in the company that sponsored the research	90	9	98	2	98	2

Participants' knowledge of the five statements posed in the case study on researchers' obligations is shown in Table 6. Participants scored higher at post-test on all but one of the five items in this case study. At pre-test, 87% of participants knew that the researcher did not protect the safety of the participants; the proportion increased to 97% at post-test. However, virtually all individuals correctly knew that the investigators did not provide adequate feedback to research participants at the three points in time (97%, 100%, and 98%).

**Table 6:** Percentage of trainees providing correct answers in case study on responsibilities of investigators

Statements	Pre (%) (N = 97)	Post (%) (N = 97)	Follow-up (%) (N = 59)
1. The women provided completely voluntary consent to participate in the study.	94	98	98
2. The payment of N100 was acceptable/appropriate as compensation for participation in the study.	86	81	78
3. The investigator provided adequate feedback to the research participants.	97	100	98
4. The researcher protected the safety of research participants in all ways.	87	97	100
5. The women who participated in this study were not exposed to any risk.	93	96	98

## Discussion

Although the number of formal and online training programs in research ethics has increased during the last five years due largely to the efforts of many regional and international organizations, many professionals in African countries still have limited access to formal training in research ethics. At the same time, more and more African scientists are involved in research, as greater numbers of clinical trials are being carried out in developing countries [6]. Even partially correcting the 10/90 gap – that 90 percent of global research is targeted at diseases comprising only 10 per cent of the global burden of disease – will contribute to more health research being conducted in developing countries. This situation underscores the need to urgently develop the capacity of a "critical mass" [16] of professionals in developing countries who have the skills to provide quality ethical oversight on these studies. Trained professionals are also required to ensure that studies conducted in developing countries respond to local health conditions, protect the rights and integrity of study participants, develop local capacity, and improve existing infrastructures [17].

The training program described in this paper was developed to contribute to fulfilling this goal. Sixty-five percent of workshop attendees had their first opportunity of formal training in research ethics as a result of their participation in this project, thus addressing an important unmet need of scientists and clinicians in this institution. The fact that trainees were drawn from a large majority (91%) of the departments/units in the institution helped to ensure that many staff were reached with this intervention. It is also encouraging that ten of the fifteen members of the local IRB participated in the training. The expectation is that the participation of these IRB members would improve their capacity to perform their oversight functions.

It is noteworthy that, during the needs assessment phase of this project none of the FGD participants considered "standard of care," "obligations of researchers to study participants," and "conflict of interest" to be relevant topics for training. It may be that the topics to which they gave higher priority were topics they have encountered more frequently through their work. These other topics, while clearly animating much of the existing research ethics scholarly literature, may be confronting these individuals less often. As knowledge of the more basic concepts becomes more sophisticated, it is likely that subsequent training covering these additional topics would be appropriate. Indeed, members of the IRB and researchers likely should have the opportunity for additional continuing education programs in research ethics to enable them to continue to develop their thinking around all of these issues as well as those that will emerge in the future.

Post-training improvements were found in participants' knowledge of the principles of research, the application of these principles, the international regulations, and the operations of an IRB. This improvement is similar to findings in previous studies [11,12] and may be attributable to the interactive nature of the workshop and facilitation by experienced resource persons. Despite initial concern that many clinicians may not have the time to attend the three-day training due to their heavy workload, many of the trainees received the opportunity for training with great enthusiasm and the majority attended all the sessions.

Overall, trainees retained much of the knowledge acquired from the workshop one month after its completion. Knowledge about the operations of IRBs and application of the principles of research improved significantly one month after completion. This may mean that trainees had greater opportunity to reflect on these issues over other topics covered. It may also imply that trainees were able to apply, or otherwise internalize, their knowledge of certain standard operating procedures of IRBs. There was only a marginal increase in trainees' knowledge relating to requirements for durational IRB protocol approvals. Perhaps this aspect of the operation of an IRB was not emphasized enough during the training. This suggests the need for the local IRB to improve its efforts at educating scientists of the fact that approvals for protocols must be renewed every year.

## Limitations

There were limitations to this study. First, the time for the follow-up was too short to determine the extent to which trainees retained the information long-term. Second, it was not designed to determine the degree to which trainees actually applied what they learned from the training. Knowledge on quizzes may or may not predict how researchers or IRBs apply these principles to their own work, and that clearly is the outcome we all strive for in conducting research ethics education. Third, lack of a comparison group limits confidence to draw firm conclusions about the outcome of the training, although it is hard to see why or how changes in responses, particularly after the one month of the workshop would be due to factors other than the training. Fourth, case studies provided only limited information to trainees and there may have been misunderstandings of what the case was describing. For example, some of the trainees who incorrectly "disagreed" that the mock study was not reviewed by an ethics committee may have done so because this information was not provided in the case study. Finally, these problems reflect the inherent limitations in using a questionnaire to test effectiveness of research ethics training. Several of the items in the questionnaire may well have assessed participants' recall of factual information about codes or definitions rather than determining whether their ability to identify or help reason through an ethics dilemma in research actually changed. Admittedly, it is more straightforward to measure knowledge. Further research identifying tools to measure sophistication of moral reasoning skills would be an important contribution to evaluation of future training efforts. One alternative may be interviews with members of the IRB and with various health researchers throughout the institution at 12 months to determine if researchers approach their research any differently in terms of ethics considerations, and if the protocols submitted by trainees have changed accordingly.

## Conclusion

Although capacity development in research ethics for researchers, members of IRB, and sponsors of research is not, alone, a guarantee of safety for research participants, it is an important component of the interventions that can contribute to that goal. Capacity development for the conduct of ethical research is required across the globe, and it is especially necessary in resource constrained settings where the risk of exploitation in research may be higher. The training program reported in this article was developed to help fulfill this goal. The training met a previously unmet need for capacity development in research ethics among academic staff in a major Nigerian university. The training improved participants' knowledge of principles of research ethics, international guidelines and regulations, and operations of IRBs, but there is an ongoing need to develop an effective mechanism for assessing the application of knowledge trainees derived from this and similar interventions.

## Competing interests

The author(s) declare that they have no competing interests.

## Authors' contributions

AJA conceived the project, developed draft instruments, supervised data collection and wrote drafts of findings. NK reviewed and edited draft instruments, supervised data collection process, reviewed and edited draft manuscripts. Both authors read and approved of final manuscript.

## Pre-publication history

The pre-publication history for this paper can be accessed here:



## Supplementary Material

Additional file 1 Pre and Post Test Questionnaire. The questionnaire is the instrument used to collect data on demographic information, knowledge about international guidelines, principles of research ethics, their application and ethics reasoning from workshop trainees. Click here for file

## References

[B1] Jeffers BR (2002). Continuing education in research ethics for the clinical nurse. The Journal of Continuing Education in Nursing.

[B2] (2002). United States of America Code of Federal Regulations.

[B3] Cohn F, Manetta A (2003). A teachable moment: research ethic revisited. American Journal of Obstetric and Gynecology.

[B4] Rosenstein DL, Miller FG, Rubinow DR (2001). A curriculum for teaching psychiatric research bioethics. Biological Psychiatry.

[B5] Sponholz G (2000). Teaching scientific integrity and research ethics. Forensic Science International.

[B6] Kilama WL (2003). Equipping Africa's researchers for global collaboration. http://www.scidev.net/dossier/index.cfm.

[B7] Research Triangle Institute (2005). Road map for success in international research: strategies for protecting human research subjects globally. Report of workshop.

[B8] Chima SC (2006). Regulation of biomedical research in Africa. British Medical Journal.

[B9] National Bioethics Advisory Commission, 2001, USA.

[B10] Council for International Organization for Medical Sciences (2002).

[B11] London L, McCarty G (1998). Teaching medical students on the ethical dimensions of human rights: meeting the challenge in South Africa. Journal of Medical Ethics.

[B12] Pollock RE, Curley SA, Lotzova E (1995). Ethics of research training for NIH T32 surgical investigators. Journal of Surgical Research.

[B13] Brown S, Kalichman MW (1998). Effects of training in responsible conduct of research: a survey of graduate students in experimental sciences. Science and Engineering Ethics.

[B14] Falusi AG Proceedings of a National Bioethics Training Workshop, 15–17, June, 2004.

[B15] Family Health International (2002). Research Ethics Training Curriculum.

[B16] Daar AS, Singer PA (2002). Human capital is key to research ethics. http://www.scidev.net/dossiers/index.cfm.

[B17] World Health Organization (2000).

