# Supplementary material for: Outcome of a research ethics training workshop among clinicians and scientists in a Nigerian university
Source: BMC Med Ethics. 2008 Jan 24;9:1. doi: 10.1186/1472-6939-9-1 (PMC2246144; doi:10.1186/1472-6939-9-1)
Supplement: Additional file 1 — Pre and Post Test Questionnaire. The questionnaire is the instrument used to collect data on demographic information, knowledge about international guidelines, principles of research ethics, their application and ethics reasoning from workshop trainees. [file 1472-6939-9-1-S1.doc]

**Pre and Post Test Questionnaire**

**Directions for Completing this Questionnaire**

Please select an identification number, symbols etc, and insert this in the space provided. Please not the same identifier should be used each time the questionnaire is completed.

Date ________________________ Identification number______________

1. Faculty _______________________ 2. Specialty ___________________
2. Highest professional/academic qualifications
   1. MSc.
   2. PhD
   3. MBBS
   4. FNCP, FWCP, FNCPH
   5. Others please specify___________________________
3. Have you ever attended any training program on research ethics?

1. Yes 2. No

1. List the three most important ethical considerations in any research project involving human participants?

1____________________________________________

2____________________________________________

3____________________________________________

1. List any three international guidelines or regulations that are supposed to guide the conduct of any research involving human participants?

1______________________________________________

2______________________________________________

3______________________________________________

7. Which of the following statement is “True” or “False” about research ethics?

|  | True  (1) | False  (2) |
| --- | --- | --- |
| 1. Only research funded by an external agency should be submitted to the Ethical Review Committee for consideration. |  |  |
| 2. Once they are enrolled, participants in a research cannot withdraw from the study without prior approval or agreement from the principal investigator. |  |  |
| 3. The information in a consent form must be presented in a manner that is comprehensible to the research participants. |  |  |
| 4. Approvals from an Ethical Review Committee are for the duration of the research. |  |  |
| 5. It is the responsibility of the researcher to develop a scientifically sound research protocol. |  |  |
| 6. The main function of an Ethical Review Committee is to ensure the protection of participants who volunteer to take part in a research. |  |  |

**Please write out in the space provided the principle of research ethics that best defines each of the following statements**

8. The capacity and rights of all potential Nigerian research participants to make their own

decision must be respected by the investigator _____________________________________

9. The special needs of vulnerable populations such as prisoners and children must be

protected at all times__________________________________________

10. The protection of the Nigerian research participants is more important than the pursuit of new

Knowledge by scientists’ ______________________________________________

11. It is the responsibility of researchers to maximize benefit and minimize risk of

all persons who take part in a research ___________________________________________

12. All segments of the Nigerian population must be fairly selected as participants in any

research project ________________________________________________________

13. The use of poor Nigerian research participants for the exclusive benefit of more privileged

Nigerians should be discouraged _______________________________________________

14. Nigerian investigators have the responsibility of ensuring the physical, mental and social

Well-being of all Nigerians who volunteer to take part in any research project. ___________________________________________________

**Please read the short stories below and answer the questions that follow.**

**Case study One**

Professor Age Madral, a renowned dermatologist in a Nigerian University, owns a large stock in ABC Drug Manufacturing Company. He is approached by ABC to conduct a study to test the efficacy of a new drug for the cure of melanonia, a type of skin disease. Madral is expected to recruit patients from his clinic into the study. Madral is excited about this study because the drug offers new hope of effective treatment for treating many of his patients with melanonia. The company is offering N2, 000 for each patient recruited in the study and a handsome honorarium to Madral for using his patients for the study. Madral agrees to collaborate and begins recruiting of patients for the study.

**15. What type of ethics concerns exists in this study? (Please circle all that apply)**

1. That Madral has a conflict of interest.
2. The study was not reviewed by an ethics review committee.
3. The amount paid to patients is acceptable or adequate.
4. **All of the above**

**16. Which of the following statements do you “agree” or “disagree” with**

|  | Agree  (1) | Disagree  (2) |
| --- | --- | --- |
| 1. Patients recruited into this study will definitely benefit from the new drug. |  |  |
| **2.** Patients who participated in this study will definitely have access to the drug if it proven to be effective. |  |  |
| 3. The amount being paid to participants as compensation is acceptable or adequate. |  |  |
| 4. Madral may experience some tension between his interests as a scientists and his interest as a share holder in the company that sponsored the research. |  |  |

**Case Study Two**

A consultant physician in a local teaching hospital is interested in studying the prevalence of sexually transmitted infections (STI) among female sex workers in Moroka town. He has identified three brothels in which his potential research participants are located. He contacted the managers of each hotel as well as the landlady (the leaders) of the sex workers and obtained approval to conduct the study. Participants in this study will be tested for 3 common STI and interviewed about their sexual behavior. Blood will be drawn and physical examination performed. He provided free STI treatment for affected women at the hospital’s STD clinic and paid N100 compensation for inconvenience and lost time. At the commencement of the study the manager summoned a general meeting of all sex workers and requested all of them to cooperate with the researchers. Although some of the women were initially reluctant to participate they agreed later because of the fear that the manager may sanction those who refuse to enroll in the study. The physician completed the study, published his findings and never returned to the hotel.

17. Which of the following statements are “True” or “False” about this study?

|  | **True**  **(1)** | **False**  **(2)** |
| --- | --- | --- |
| **1. The women provided completely voluntary consent consented to participate in the study.** |  |  |
| 2. The payment of N100 was acceptable /appropriate as compensation for participation in the study. |  |  |
| 3. The investigator provided adequate feedback to the research participants. |  |  |
| 4. The researcher protected the safety of research participants in all ways. |  |  |
| 5. The women who participated in this study were not exposed to any risk. |  |  |
